# Moving Cages Further Offshore: Effects on Southern Bluefin Tuna, *T. maccoyii*, Parasites, Health and Performance

**DOI:** 10.1371/journal.pone.0023705

**Published:** 2011-08-25

**Authors:** Nicole T. Kirchhoff, Kirsty M. Rough, Barbara F. Nowak

**Affiliations:** 1 National Centre for Marine Conservation and Resource Sustainability, University of Tasmania, Launceston, Tasmania, Australia; 2 Australian Southern Bluefin Tuna Industry Association, Port Lincoln, South Australia, Australia; Institute of Marine Research, Norway

## Abstract

The effects of offshore aquaculture on SBT health (particularly parasitic infections and haematology) and performance were the main aim of this study. Two cohorts of ranched Southern Bluefin tuna (SBT) (*Thunnus maccoyii*) were monitored throughout the commercial season, one maintained in the traditional near shore tuna farming zone and one maintained further offshore. SBT maintained offshore had reduced mortality, increased condition index at week 6 post transfer, reduced blood fluke and sealice loads, and haematological variables such as haemoglobin or lysozyme equal to or exceeding near shore maintained fish. The offshore cohort had no *Cardicola forsteri* and a 5% prevalence of *Caligus* spp., compared to a prevalence of 85% for *Cardicola forsteri* and 55% prevalence for *Caligus* spp. near shore at 6 weeks post transfer. This study is the first of its kind to examine the effects of commercial offshore sites on farmed fish parasites, health and performance.

## Introduction

Offshore aquaculture is in its infancy worldwide, yet commercial development is underway in numerous countries including USA, Ireland, Norway, Spain, Italy, Malta, Belgium, Scotland, UK, Japan, and Australia [1]. There are numerous factors which distinguish between a near shore and an offshore site, including location or hydrography, the in-water and above-water environment, the ease of access and associated operation logistics, yet no formal international definition has been made. In the context of this study, offshore was defined by reduced access (i.e. remoteness) and increased exposure to the environment, both in- and above-water. The attractiveness and potential benefits of moving aquaculture cages further from the shore are many, including fewer limits to the scale of operation, enhanced water quality, lower costs of environmental monitoring, reduced interaction with urban populations and inshore environmental concerns, and reduced disease risk [2]. In addition, moving cages from near shore to offshore sites may be necessary in the future due to the many anticipated effects of climate change [3]. Yet, many of these assertions have been insufficiently tested and the commercial feasibility of offshore development is presently unknown.

Offshore aquaculture has not been extensively developed for many reasons. Moving farther offshore is capital intensive, leading to increases in operation and servicing costs which need to be outweighed by potential performance benefits of the cultured species. There are also investment uncertainties related to the optimal configuration of sites, species most suitable to the exposed conditions, and lack of necessary technology [1]–[2]. Technology does not just refer to strong farming structures cages; it also concerns advanced feeding techniques, communication, mortality retrieval systems, and monitoring systems, which allow management of stocks that are not easily accessible [1]. In addition, sufficient testing of the feasibility of offshore aquaculture also requires large baseline datasets in full commercial scale, which are absent in the current published literature.

Southern Bluefin Tuna (SBT) have been ranched in near shore cages in Port Lincoln, South Australia since 1991. In Australia, schools of 2–4 year old wild SBT are captured by purse seine and, carefully towed back to the Tuna Farming Zone (TFZ) in Spencer Gulf near Port Lincoln, South Australia where they are transferred into several grow-out cages and fattened on baitfish for three to six months. As a member nation of the Commission for the Conservation of the Southern Bluefin Tuna (CCSBT) and with sustainability in mind, the Australian SBT industry strictly adheres to catch quotas, quantified upon the arrival of a tow cage within the TFZ, prior to the start of ranching. Large commercial scale baseline datasets have been collected for several decades concerning environmental monitoring, stock performance and health, and economic viability of ranched SBT maintained in the TFZ (Australian Southern Bluefin Tuna Industry Association (ASBTIA) pers. comm.), enabling future research into alternative husbandry practices, such as site. In addition, current private investments made by the Australian SBT ranching industry into technology and operations infrastructure can be easily translated to the offshore environment. The aim of this project was to examine the feasibility of offshore versus near shore aquaculture using the ranching of SBT in Port Lincoln, South Australia as a case study. In this study, feasibility was measured through SBT health, i.e. parasite loads and haematology, and performance, i.e condition index and survival. Although cost cannot be directly considered in this study due to commercial confidentiality restrictions, economic implications are discussed.

## Materials and Methods

### Ethics Statement

All work with animals, samples and methods for recovering samples were approved by the University of Tasmania board of animal ethics, project number A0010593.

### Experimental Fish and Site Characteristics

**Figure 1:**
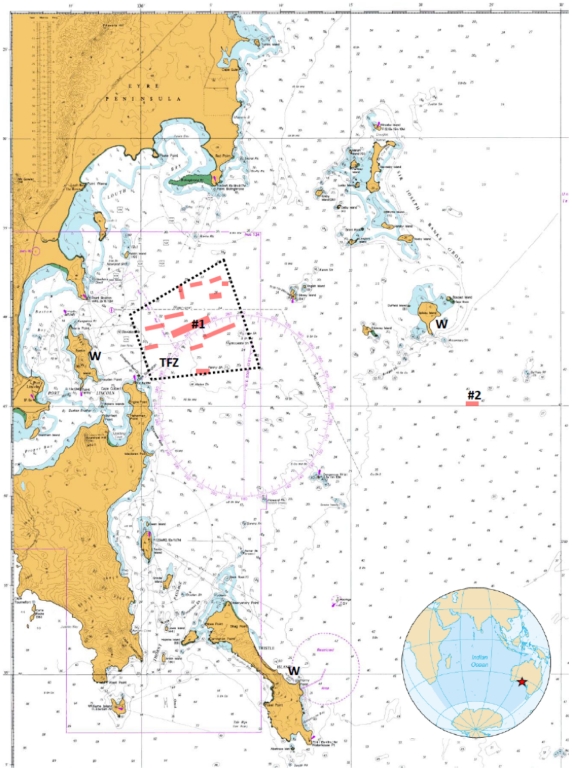
Map of Boston Bay in the south-west Spencer Gulf indicating commercial lease sites in red. Also noted are the near shore cage study site (#1) and offshore cage site (#2), and the 2010 Tuna Farming Zone in dotted box. ‘W’ denotes weather stations used for wind speed measurements.

Two different cohorts of SBT were captured by purse seine in the Great Australian Bight in February 2010. Each cohort was transported to the (TFZ) in a separate towing cage. The near shore cohort of 9165 SBT was transferred on 14/3/2010 into three grow out cages and the offshore cohort of 7300 SBT was transferred on 15/3/2010 into three grow out cages. The near shore site was located at 34° 40.299’ S, 136°04.708’ E and the offshore site was located at 34° 44.409’ S, 136°22.703’ E (Figure 1). A complete description of the hydrology other environmental parameters for each site can be found in Table 1. Transfer procedures were identical between both sites, carefully stipulated and monitored by the Australian Fisheries Service as part of the CCSBT quota allocation guidelines. SBT were stocked at an initial cage density of 3.32 kg m^−3^ for the near shore cohort and 3.37 kg m^−3^ for the off shore cohort. SBT were fed frozen sardines at an average rate of 0.8 kg SBT^−1^ day^−1^ for their entire ranching period. In 2010, commercial sites within the near shore TFZ ranged in size between 63 and 341 ha, with an average biomass at harvest of 2,283 kg ha^−1^. The whole near shore TFZ is 17200 ha, of which 1569.5 ha was under commercial lease in 2010. The commercial offshore site was 100 ha, with an average biomass at harvest of 3,087 kg ha^−1^. In 2010 the whole offshore site was 38350 ha.

**Table 1:** Comparison chart of the remoteness, above water environment and hydrology of the two farming sites, near shore in the Tuna Farming Zone (TFZ) and offshore.

Description Parameter		Near shore (TFZ)	Offshore	Reference
Remoteness	Distance from shore	16nm	25nm	
	Average number days per week site could be accessed by vessel	6–7 days	4–6 days	
Above water environment	Average wind speed	8.96knots	11.22knots	4
	Maximum wind speed	32.53knots	36.85knots	4
Hydrology	Water depth	20m	40m	
	Maximum significant wave height	0.8m	1.5–2m	5
	Tidal flow at 3.2m above seafloor	*≤*0.1m s-1	>0.5m s-1	6–7
	Flushing rate	2 days	<6hours	6
	Number of days per year sediments in top 10m of water column	5–10	<1	5
	Sediment environment	Depositional	Erosional	5

Distance from shore is represented as distance from port. Wind Speeds were measured using weather stations at Boston Island for the near shore site and an average of stations at Spilsby Island and Thistle Island (Figure 1).

### Sample Collection

#### Field Collection

Ranched SBT are wild and each cohort consists of several schools of SBT mixed together, therefore individual SBT were used as replicates for this study, not cages of SBT, to measure the effect of ranching site on SBT. Sampling was limited due to the high commercial value of the fish, limiting total sample size to 100 SBT. Three sampling time points of samples were chosen for this study: at transfer into grow out cages to demonstrate initial differences between the cohorts of fish, at week 6 of ranching duration to demonstrate effects of site on ranching performance and at week 23 to demonstrate effects of site on long term ranching performance. Week 6 was chosen as the most important sampling time point for two reasons: (1) limited effects of captivity on ranched SBT prior to 6 weeks have been observed and (2) a significant mortality event and health changes are known to occur at week 6 in near shore ranched SBT [8]. Samples were collected from both the near shore and offshore SBT at transfer (n = 10 per site), week 6 (n = 20 per site) and week 23 at harvest (n = 20 per site) post transfer. Transfer samples were collected during transfer from the tow cage to the grow-out cage and the week 6 and week 23 samples were collected during commercial harvests. At the initial and week 6 time points, SBT were sampled using a baited hook and line. Divers caught the SBT at the 23 week sampling. The total time between capture and killing of each SBT was less than one minute for both catching methods. Once on the boat, SBT were immediately spiked in the head, the brain was removed using a ‘Taniguchi tool’ (core), and a wire was placed down the spine to destroy the spinal nerves. Length and weight was recorded for all SBT at the time of sampling. At transfer and week 6, SBT were weighed whole, but at week 23 SBT were weighed after the gills and viscera were removed due to space limitations associated with large commercial harvests. Weight for SBT sampled in week 23 was corrected by dividing weight (kg) by 0.87 [9]. Condition index was calculated for each sample using the formula: weight (kg) divided by length (m)^3^. Immediately after external surface examination, whole blood was collected from the severed lateral artery in the pectoral recess in two 10 ml tube (Sarstedt, Ingle Farm, South Australia), one heparinized and one non-heparinized, and placed on ice. Blood was collected within 3 minutes of fish capture. During transfer and week 6 sampling, parasites were quantified. External metazoan parasites were quantified from both the skin and gill arches using the naked eye during killing or as soon as possible after the SBT were killed. All lice visible to the naked eye were collected as soon as possible; any additional lice remaining on tuna surfaces were then detected using a technique described previously [9]. Parasites were not quantified from the week 23 samples as previous studies have determined parasites loads to peak on ranched SBT earlier in the ranching season [10]. The gills and viscera were then excised. The heart was placed in a waterproof tub, the visceral organs were placed in a waterproof bag and both stored on ice.

#### Laboratory Processing

The heparinized vial of whole blood was used for whole blood and plasma aliquots. Three 500 µl aliquots of whole blood were transferred into 1.5 ml plastic tubes and frozen at −20°C. The remaining blood was centrifuged at 3000 xg at 4°C for 5 minutes. Blood plasma was aliquoted into five-1.5 ml plastic tubes, and frozen at −20°C. The non-heparinized vial of whole blood was used for serum collection. Vials were stored upright at 4°C for 24 hours, centrifuged at 1000 xg at 4°C for 5 minutes, and serum aliquoted into three 1.5 ml tubes. Serum samples were stored at −20°C. Hearts were dissected 2–4 h after removal from the carcass and flushed with physiological saline to dislodge any adult *Cardicola forsteri* [see 11]. Flushes were then poured into Petri dishes and examined for the presence of adult *C. forsteri* using a dissection microscope.

#### Blood Variables: Hematology

Haemoglobin concentrations were determined from whole blood aliquots using the cyanometahaemoglobin assay based on [12] modified by [13].

Blood plasma glucose and lactate were measured using Accu-Chek® Advantage II and Accutrend ® Plus by Cobas, respectively. The pH of blood plasma samples was measured using a Minilab Isfet pH meter Model IQ125 (IQ Scientific, USA). Blood plasma osmolality was determined using a Vapro© Model 5520 vapour pressure osmometer (Wescor Inc., Logan, Utah, USA).

#### Blood Variables: Humoral Immune Response

Blood serum was analyzed in triplicate for lysozyme activity and alternative complement activity. Lysozyme activity was measured using a method based on that described by [14] modified by [13]. Blood serum alternative complement activity was measured using a modified [15] method as described by [13].

#### Statistical analyses

Parasite infections were characterized by prevalence (the number of host infections as a proportion of the population at risk), mean intensity (the average number of parasites per infected host) and mean abundance (the average number of parasites in all hosts) [16]. Sterne's exact 95% confidence intervals were calculated for prevalence, and 95% bootstrap confidence intervals (with 2000 replications) were calculated for mean abundance, using the software ‘Quantitative Parasitology 3.0’, supplied by [17]. The prevalence and mean abundance for each species were compared between treatments and ranching durations in a pairwise fashion. Given the high total number of pairwise comparisons, α  =  0.01 was regarded as significant for these statistics.

All other performance, haematology, and immunology results were interpreted using the R 2.8.1 statistical package (© 2008, The R Foundation for Statistical Computing). Survival was assessed using log-rank test for equality of the two Kaplan-Meier survival curves, one for each treatment. Condition index and blood parameters were analyzed for differences between each treatment at each sample date, and differences within a treatment for all sample dates using ANOVA. The assumption of homogeneity of variances was checked by the residual plot and Bartlett test and variables transformed when necessary. The Tukey HSD post-hoc test was applied at a significance level of α = 0.05, to determine differences between the explanatory variables. Plasma pH was log 10 transformed due to failure to pass the Bartlett test for normalcy.

## Results

While the offshore cohort began ranching (i.e. at transfer) with a lower condition index (F = 5.7614, df = 1,18, p = 0.0274), their condition increased considerably between transfer and week 6 of ranching (Table 2). At week 6, the offshore cohort averaged higher condition index compared to the near shore cohort (F = 5.5738, df = 1,38, p = 0.0235). The offshore cohort maintained condition index from week 6 to week 23 of ranching (p>0.05), while the near shore cohort continued to increase to a condition equal to the offshore cohort by week 23 (F = 0.569, df = 1,37, p = 0.4554). Changes in condition index can be expected to occur in an asymptotic fashion during fattening, quickly increasing from low to medium conditions and increasing much slower at higher condition.

**Table 2:** Mean ± SE for length (cm), weight (kg), condition index and blood parameters in ranched SBT at transfer, week 6 and week 23 of ranching in the near shore and off shore cohorts.

	Near shore			Offshore		
	Transfer	6 weeks	23 weeks	Transfer	6 weeks	23 weeks
**Length**	96.9±1.6	114.3±2.6	117.8±1.7	99.0±2.7	116.9±2.8	122.2±2.1
**Weight**	17.9±1.0	32.6±1.9	38.4±1.5	18.4±1.5	37.3±2.1	42.3±2.0
**Condition Index**	19.45±0.34^a^	21.63±0.43^b^	23.27±0.32^c^	18.50±0.21^a^ *	23.08±0.44^b^ *	22.96±0.27^b^
**Hb**	19.40±0.50^ab^	20.33±0.37^a^	18.84±0.27^b^	18.95±0.43	19.59±0.50	20.12±0.34*
**pH**	7.87±0.05^b^	8.09±0.03^ a^	7.66±0.03^c^	7.96±0.05^a^	8.04±0.02^a^	7.62±0.03^b^
**Osmolality**	394.2±4.5^a^	412.6±4.5^b^	451.5±4.6^c^	388.4±4.5^a^	421.4±5.1^b^	477.5±6.9^c^ *
**Glucose**	3.03±0.29^a^	5.79±0.35^b^	6.22±0.16^b^	3.65±0.74^a^	6.39±0.37^b^	6.58±0.27^b^
**Lactate**	10.16±0.29^ab^	11.09±0.39^a^	9.59±0.29^b^	6.72±0.70^a^ *	10.22±0.27^c^	8.73±0.49^b^
**Lysozyme**	71.69±27.56^ab^	151.03±27.56^a^	73.04±14.54^b^	77.20±25.71^a^	202.25±25.71^b^	55.34±14.17^a^
**ACH50**	184.38±23.81^a^	119.56±10.30^b^	85.28±6.08^c^	236.54±65.91^a^	141.79±14.75^b^	82.35±13.03^b^

Harvest weights are corrected for gg weight collection. Blood parameters include haemoglobin (Hb) (g dL^−1^), plasma pH, plasma osmolality (mmol kg^−1^), plasma glucose (mmol L^−1^), plasma lactate (mmol L^−1^), lysozyme (ug mL^−1^), and alternative complement (ACH50) (units mL^−1^) activity. Different letters denote significant differences over time within each treatment. * denotes statistical differences between the offshore cohort and the near shore control cohort.

**Figure 2:**
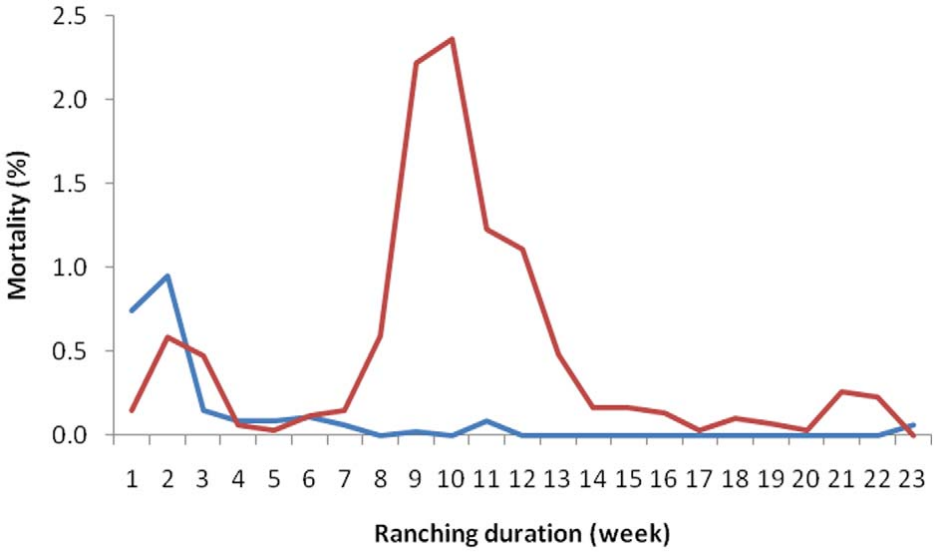
Cumulative mortality in ranched SBT in the near shore (denoted in red) and off shore cohorts (denoted in blue). There was a difference in survival curves over the first twelve weeks of ranching (χ^2^ =  107, df = 1, p<0.001).

The offshore cohort had higher survival through the ranching period (χ^2^ =  107, df = 1, p<0.001) (Figure 2), with at 5.6% cumulative mortality compared to 10% of the near shore cohort. Initial mortality was higher in the offshore cohort, and may be attributed to different conditions on the tow from the capture site to the lease site or due to initial unfavorable conditions at the offshore lease site early in the ranching season. Nearly 80% of the total mortality in the offshore cage occurred in week 1 and 2 of ranching. The near shore cohort had little initial mortality, with 84% of the total mortality occurring between week 8 and 12 of ranching. An approximate 100 SBT were unaccounted for in the weekly mortality counts for the offshore cohort. Upon consultation with the farm manager, it was assumed these fish were victim to either shark attacks or poaching, although neither assumption can be confirmed. Cumulative mortality in the offshore cohort was 2.5% when the unaccounted SBT were not included in the calculation.

At week 23 of ranching, the offshore cohort had 1.5 g dL^−1^ higher haemoglobin concentration (F = 15.920, df = 1,38, p<0.001) and 20 mmol kg^−1^ higher osmolality (F = 9.7547, df = 1,38, p = 0.003) compared to the near shore cohort (Table 2). While blood plasma lactate was higher in the offshore cohort at transfer (F = 20.592, df = 1,18, p<0.001), it was not different at week 6 or week 23 of ranching (Table 2). Therefore the initial difference may be attributed to differences between the cohort in tow and/or transfer conditions, not effects of location. No other differences were found in blood parameters or performance between cohorts (p>0.05) (Table 2).

Offshore fish had lower prevalence (p = 0.048) and mean abundance (t = −2.366, p = 0.0235) of *Caligus* spp at 6 weeks of ranching compared to near shore SBT. While offshore SBT maintained low *Caligus* infections from transfer to week 6, the prevalence in the near shore cohort increased from 0 to 55% (p = 0.004) and the mean abundance increased from 0 to 0.65 *Caligus* per fish (t = 3.901, p = 0.0045) (Table 3). There was no *Cardicola forsteri* infection within the offshore cohort between transfer and week 6 of ranching. Prevalence of *C. forsteri* in the near shore cohort increased from 20 to 85% (p = 0.001), mean intensity from 1 to 4.18 flukes (t = 4.452, p = 0.001), and mean abundance from 0.20 to 3.55 (t = 4.741, p = 0.001) over the same time period (Table 3). No differences in prevalence, mean intensity, or mean abundance of *Caligus* sp or *Cardicola forsteri* were found between the two sites at the start of ranching (i.e. at transfer). There was no effect of sampling date or location on the mean intensity of *Caligus* sp.. No differences were found in prevalence, mean intensity, or mean abundance of gill parasites (*Hexostoma thynni, Pseudocycnus appendiculatus,* and *Euryphorus brachypterus*) (Table 3).

**Table 3:** Parasite prevalence (P) (95% confidence interval), mean intensity (I) (95% confidence interval) and mean abundance (A) (95% confidence interval) in ranched SBT at transfer and week 6 of ranching in the near shore and off shore cohorts.

	Near shore					
	Transfer			Week 6		
	P (%)	I	A	P (%)	I	A
***Caligus*** ** spp.**	0.0 (0.0-29.1)^b^	0.0 (na)	0.00 (0.00-0.00)^b^	55.0 (32.0-75.6)^a^	1.2 (1.0-1.6)	0.65(.35-1.00)^a^
***Hexostoma***	10.0 (.5-44.6)	1.0 (0.0-1.0)	0.10 (0.00-0.30)	15.0 (4.2-37.2)	2.0 (1.0-2.7)	0.30 (0.00-0.75)
***Pseudocycnus***	40.0 (15.0-70.9)	3.0 (1.5-5.0)	1.20 (0.30-1.80)	15.0 (4.2-37.2)	1.0 (0.0-1.0)	0.15 (0.00-0.30)
***Euryphorus***	20.0 (3.7-55.4)	2.5 (1.0-2.5)	0.50 (0.00-1.70)	25.0 (10.4-47.5)	1.2 (1.0-1.4)	0.30 (0.10-0.55)
***Cardicola forsteri***	20.0 (3.7-55.4)^b^	1.0 (na)^b^	0.20 (0.00-0.40)^b^	85.0 (62.8-95.8)^a^	4.2 (3.1-5.8)^a^	3.55 (2.35-4.95)^a^

	Offshore					
	Transfer			Week 6		
	P (%)	I	A	P (%)	I	A
***Caligus*** ** spp.**	10.0 (0.5-44.6)	1.0 (0.0-1.0)	0.10 (0.00-0.30)	20 (7.1- 42.35)*	1.0 (0.0-1.0)	0.20 (0.05-0.35)*
***Hexostoma***	0.0 (0.0-29.1)	0.0 (na)	0.00 (0.00-0.00)	20.0 (7.1-42.4)	1.8 (1.0-2.5)	0.35 (0.10-1.05)
***Pseudocycnus***	20.0 (3.7-55.4)	4.0 (1.0-7.0)	0.80 (0.00-2.90)	5.0 (0.3-24.4)	2.0 (0.0-2.0)	0.10 (0.00-0.30)
***Euryphorus***	30.0 (8.7-61.9)	1.0 (0.0-1.0)	0.30 (0.00-0.50)	25.0 (10.4-47.5)	3.6 (1.8-5.4)	0.90 (0.25-2.05)
***Cardicola forsteri***	0.0 (0.0-29.1)	0.0 (na)	0.00 (0.00-0.00)	0.0 (0.0-16.7)*	0.0 (na)*	0.00 (0.00-0.00)*

Different letters denote significant differences over time within each treatment. * denotes statistical differences between the offshore cohort and the near shore control cohort.

## Discussion

SBT maintained offshore had better survival, lower *Caricola forsteri* and *Caligus* parasite loads, and the hematology of SBT ranched offshore was equal to or exceeding SBT maintained in the traditional near shore ranching environment. These results suggest the offshore cohort may be able to respond better to ranching compared to SBT maintained near shore possibly due to better environmental conditions.

The observation of improved survival within the offshore cohort is the most significant outcome of this experiment. An average 6–14% cumulative mortality has been reported across the industry, occurring mostly in a restricted period from 6 to 12 weeks of ranching [8]. This annual mortality event has been observed within ranched SBT in South Australia since 1997 (ABSTIA pers. comm.) and was also observed within the near shore cohort of SBT in this study. The timing of this mortality, the duration of the event, and its severity is observed to vary annually [18], between tows [13], [18], by the timing of tow arrival within a season, between companies or husbandry techniques [18], and even between cages within the same tow [9], [13]. Because the cause of the annual mortality event is unknown, it cannot be conclusively stated whether or not the offshore cohort maybe impacted in the future. Yet, current results suggest maintaining fish offshore may prohibit exposure of fish to the near shore mortality event, therefore maintaining enhanced survival in the future. A future study is currently underway to determine if temporary offshore holding can offer similar benefits to survival through to harvest.

The offshore cohort also demonstrated enhanced condition earlier in the season. Although the offshore cohort had a lower condition index at the beginning of ranching than the near shore cohort, they quickly gained in condition, both surpassing the near shore cohort and obtaining a condition equivalent to harvest quality by week 6 of ranching. The ability to have SBT reach harvest condition as early as possible in the ranching season is advantageous for the Australian commercial SBT operation, as it allows them to market fish earlier in the season for the fresh market. Not only does each SBT sold on the fresh market obtain a higher market value as opposed to the frozen market at the end of the season, but early stock harvests reduce feeding and maintenance costs (ASBTIA pers comm.). The observed enhanced condition may be attributed to the lack of stress and an improved ability to convert feed into growth, and/or may demonstrate an improved ability to acclimate to ranching more quickly than those SBT maintained near shore.

Another promising result of offshore SBT ranching is a reduction in sealice and blood fluke infections. Described in 1997 [19], a blood fluke, *Cardicola forsteri* is currently a common and prevalent infection in ranched SBT [9], [11], [13], usually infecting up to 100% of ranched SBT after two months of captivity [11]. In 2004, *C. forsteri* was identified as one of the most significant risks associated with Australian ranched SBT [20], therefore reduction of this infection is an important result for the commercial industry. It is possible that the greater depth and current velocity may offer protection against infection by decreasing the incidence of cercariae within the cages as the intermediate host known to be a benthic terebellidae polycheate, *Longicarpus modestus* [21]. It may also be possible that the intermediate host is absent from the offshore site as its distribution is not known. Finally, enhanced health condition of the offshore SBT may also reduce infection success. Ranched SBT are able to develop a specific antibody response against *C. forsteri* and reduced infection burdens have been observed over ranching duration [22]. Research is currently underway to determine the trigger for specific antibody production to *C. forsteri* and its effects against current infection and future exposure. Further research is needed on the biology and distribution of the intermediate host and behavior and biology of the cercariae to assess a ranching site's risk for *C. forsteri* infection.

The lack of *Cardicola forsteri* infection observed within the offshore cohort provides a unique opportunity to investigate some of the claims of *C. forsteri* induced performance and health effects on SBT. It has been commonly assumed all ranched SBT maintained at the traditional near shore location are exposed to *C. forsteri* cercariae as soon as they enter the farming zone [11]. In the past researchers were not able to uncouple the effects of captivity from the effects of infection due to the 100% prevalence of *C. forsteri* within ranched SBT. It has been suggested *C. forsteri* infection may cause a reduction in haemoglobin concentration [9], an elevation in lysozyme concentration [8]–[9], and an elevation in alternative complement activity [8]. While there was no *C. forsteri* within the offshore cohort compared to 85% prevalence within the near shore cohort at week 6 of ranching, there was no difference between cohorts in haemoglobin concentrations or humoral immune response. It is therefore unlikely that the observed mean intensity of infection with *C. forsteri* induces a haemoglobin reduction or changes in humoral immune response, although infection intensities found in the near shore cohort were low and highly variable which may mask or prevent significant haematological differences. It may also be possible that the effect on haemoglobin or humoral immune response to *C. forsteri* is short-lived [8], therefore may have been missed by the large gaps between sampling times in this study. Lysozyme was significantly elevated in both cohorts at week 6, despite the absence of *C. forsteri* in the offshore group at this time. Complement activity progressively declined in both cohorts, despite the significant increase in *C. forsteri* prevalence and abundance in the near shore cohort and the absence of this parasite offshore. It has been suggested humoral immune response increases with duration of ranching [23], yet this trend was not observed within this study or within other previous studies [8], [13]. Previous research has found no association between *Cardicola forsteri* infections and performance of SBT, measured as condition index and mortality [11]. Yet, enhanced performance in the offshore cohort during the first few months of ranching may suggest a link which should be further investigated. There was also a reduction in mortality from week 6 to 12 of ranching, consistent with the suggestion that *C. forsteri infection* may be associated with mortality [8]–[9], [13]. However, lower parasitic infections and reduced mortality in offshore SBT maybe a spurious relationship. Our results do not provide scientific evidence for the role of *C. forsteri* in SBT mortality or health effects due to the large number of differences between the offshore and the traditional near shore ranching environments and the limited number of sampling dates. However, this study does propose a potential role for offshore maintained SBT as a control group for future investigations into the effects of *C. forsteri.*

A lower infection of *Caligus* spp. was observed within the offshore cohort. An epizootic of *Caligus* spp. on ranched SBT is also a common and prevalent infection [20]. Prevalence increased from 0% at transfer to 55% at week 6 in the near shore cohort, consistent with previous descriptions of ranched SBT infection [10], [24]. *Caligus* spp. prevalence has been shown to decline from week 6 onward so that by week 18 infection was largely absent from the ranching population [10], [13], [24]. In contrast to *Caligus* infections in other farmed fishs, larval stages are rarely detected on ranched SBT, indicating Deagan's leatherjacket as an alternative source of mobile adult *Caligus* infections [25]. The reservoir of *Caligus*, Degen's leatherjacket (*Thamnaconus degeni*) [9], which are commonly attracted to the SBT grow-out cages during feeding [10], [24], [26]. These fish are benthic scavengers, and it has been suggested moving SBT into deeper water may reduce interactions between SBT and the source of the *Caligus*, therefore reducing infection rates [26]. It is unknown if location differences alone can explain the decline in *Caligus* infection as husbandry differences may also be attributed. Enhanced feeding protocols may also reduce the attractiveness of the cages to opportunistic feeding by demersal fish. There is a relationship between the mean intensity of *Caligus* spp. infection and severity of eye damage as well as decreased condition index [9]–[10], [24]. The offshore cohort had both reduced prevalence and abundance of *Caligus* spp. and enhanced performance, i.e. condition index, although a causal link between the two findings cannot be made. Again, no differences were found between near shore and offshore maintained fish in humoral immune response, suggesting no effect of *Caligus* infection. Although infection intensities observed within this study were low.

There were two further differences in haematology of the offshore cohort compared to the near shore cohort: osmolality and haemoglobin concentration. Elevated osmolality was observed in offshore fish at week 23. Blood osmolality is known to increase when marine fish are not osmoregulating properly, for example at times of handling and transport [27]–[29]. During SBT end of ranching harvest procedures, fish are corralled into a restricted area, the increased fish density making diver associated harvest quicker and therefore more humane for the fish. Since no changes were observed in other stress-associated parameters of blood lactate and glucose concentrations, it is likely this corralling event just prior to harvest caused the elevated osmolality and not a long-term effect of ranching site. The offshore cohort was found to maintain stable haemoglobin levels throughout the ranching season unlike the near shore cohort in which haemoglobin concentration was first elevated and then decreased between week 6 and week 23 of ranching. While the changes in haemoglobin concentration observed within this study occurred at only one time point and the magnitude may seem physiologically insignificant, previous studies have determined changing haemoglobin levels are associated with the mortality event in near shore fish [8]. Therefore the maintenance of stable haemoglobin levels in the offshore fish may be further evidence these fish were not effect by the near shore mortality event and may be further evidence of better health and wellbeing of the offshore cohort compared to the near shore cohort.

Completing this study within the restrictions of commercial operations caused the experimental design to be unavoidably compromised in two ways: restricted sample size and two discrete cohorts were used for comparison. Samplle size was maximized by limiting sample time points to those previously observed to yield the greatest significance. In addition, the effects of ranching site was discussed not only in comparison to fish ranched within the traditional near shore TFZ within the same season, but with historical data collected over several years. Although different cohorts of fish may react differently to ranching [13], the parasite load, performance, and haematology observed in the offshore SBT was drastically different than expected variance between near shore maintained cohorts [8], [13], therefore the importance of the findings within this study remain significant to the literature and our understanding of the effects of offshore finfish culture.

This is the first time the feasibility of offshore SBT ranching has been demonstrated on a commercial scale. A reduction in mortality and a reduction in the duration of fattening required to obtain harvest condition may outweigh the increased operation costs, therefore making the move offshore economically viable. In addition, numerous benefits of offshore culture including reduced blood fluke and sealice loads and haematological health equal to or exceeding near shore maintained fish may validate moving further offshore from an animal welfare point-of-view.
